# Randomized Placebo‐/Sham‐Controlled Trials of Spinal Cord Stimulation: A Systematic Review and Methodological Appraisal

**DOI:** 10.1111/ner.13018

**Published:** 2019-07-15

**Authors:** Rui V. Duarte, Ewan McNicol, Luana Colloca, Rod S. Taylor, Richard B. North, Sam Eldabe

**Affiliations:** ^1^ Liverpool Reviews and Implementation Group University of Liverpool Liverpool UK; ^2^ Department of Pharmacy Practice MCPHS University Boston MA USA; ^3^ Department of Pain Medicine Atrius Health Boston MA USA; ^4^ Department of Pain and Translational Symptom Science, School of Nursing University of Maryland Baltimore MD USA; ^5^ Department of Anesthesiology and Psychiatry, School of Medicine University of Maryland, Baltimore, University of Maryland Baltimore MD USA; ^6^ Center to Advance Chronic Pain Research University of Maryland Baltimore MD USA; ^7^ Institute of Health and Well Being University of Glasgow Glasgow UK; ^8^ College of Medicine and Health University of Exeter Exeter UK; ^9^ Neurosurgery, Anesthesiology and Critical Care Medicine (ret.) Johns Hopkins University School of Medicine Baltimore MD USA; ^10^ Department of Pain Medicine The James Cook University Hospital Middlesbrough UK

**Keywords:** Placebo, randomized controlled trials, sham, spinal cord stimulation, systematic review

## Abstract

**Objectives:**

The recent availability of paraesthesia/sensation free spinal cord stimulation (SCS) modalities allow the design of clinical trials of SCS using placebo/sham controls and blinding of patients, clinicians, and researchers. The aims of this study were to: 1) systematically review the current evidence base of randomized controlled trials (RCTs) of SCS placebo/sham trials and 2) to undertake a methodological critique of their methods. Based on this critique, we developed a checklist for the design and reporting of future RCTs of SCS.

**Materials and Methods:**

Electronic data bases were searched from inception until January 2019 for RCTs of SCS using a placebo/sham control. RCTs with only an active comparator arm were excluded. The results are presented as a narrative synthesis.

**Results:**

Searches identified 12 eligible RCTs. SCS modalities included paraesthesia stimulation, subthreshold, burst, and high‐frequency SCS and were mainly conducted in patients with failed back surgery syndrome, complex regional pain syndrome, and refractory angina. The quality and transparency of reporting of the methods of placebo stimulation, blinding of patients, clinicians, and researchers varied markedly across studies.

**Conclusions:**

To date the methods of placebo/sham control and blinding in RCTs have been poorly reported, leading to concerns about the validity and replicability of the findings. Important aspects that need to be clearly reported in the design of placebo‐/sham‐controlled RCTs of SCS include the transparent reporting of stimulation programming parameters, patient position during perception threshold measurement, management of the patient handheld programmer, frequency of recharging, and assessment of the fidelity of blinding.

## INTRODUCTION

High‐quality randomized controlled trials (RCTs) are considered the gold standard to evaluate the effectiveness of a medical treatment 1. The importance of placebo and its potential application in research studies has been recognized since 1955 2. Placebo or sham (referred to as placebo for the remainder of this manuscript) controlled RCTs are common when evaluating the efficacy of drugs 3. Furthermore, it has been observed that the brain's neurochemical activity changes when there is a belief or expectation of treatment outcomes 4. It is widely accepted that use of a placebo control in a clinical trial can reduce bias as the result of the unblinding (knowing the treatment received) of patients, clinicians, and researchers can result in reporting bias and nonspecific treatment effects reported by patients. Nevertheless, in contrast to drug therapies, providing an appropriate placebo control in clinical trials of healthcare procedures involving a medical device is often much more challenging. In addition, the daily interaction of patients with a programmable implanted device may differ from that of drug intake 5.

Spinal cord stimulation (SCS) is a recognized option for the management of several chronic pain conditions, and RCTs have been performed to investigate its effectiveness for failed back surgery syndrome (FBSS) 6, complex regional pain syndrome (CRPS) 7, painful diabetic neuropathy 8, and refractory angina (RA) 9. Some part of the pain relief observed at early stages of SCS therapy may be the result of a placebo effect with long‐term follow‐up revealing loss of efficacy for a proportion of patients when compared to the primary endpoint 10, 11, 12, 13, 14.

The design of most RCTs of SCS to date have been “open label,” that is, with an active comparator most commonly a form of conventional medical management. Furthermore, because of the paraesthesia associated with traditional SCS, it has not been possible to blind patients. However, a number of new sensation free SCS modalities are now available such as burst, high frequency, or higher density. The emergence of these new modalities has led to the conception of placebo RCTs in this field of research. Despite blinding difficulties, conventional or paraesthesia producing SCS has been compared to sham stimulation in a number of small studies with varied results, including the effects of sham stimulation being similar to those of active treatments 15, 16.

With the advent of a new paradigm for the comparator arm in RCTs to investigate the effectiveness of SCS, it is important to assess the methods used to date to facilitate placebo neurostimulation.

The aim of this systematic review was to assess the modalities, settings, and general management of participants' equipment in a placebo comparator arm in RCTs of SCS. We discuss potential issues associated with the different methods and provide a model for future RCTs in this area.

## METHODS

The systematic review methods followed the general principles outlined in the Centre for Reviews and Dissemination (CRD) guidance for conducting reviews in health care 17. This systematic review is reported in accordance with the Preferred Reporting Items for Systematic Reviews and Meta‐Analyses (PRISMA) 18. The protocol for this review is registered on PROSPERO as CRD42018090412. The current review focuses on methodological aspects of RCTs of SCS placebo‐controlled trials.

### Search Strategy

Electronic data bases MEDLINE, CENTRAL, EMBASE, and WikiStim were initially searched from inception until February 2018 and updated on January 29, 2019. The search strategies were designed using a combination of both indexing and free text terms with no restriction on language. The search strategy used for the MEDLINE data base is presented in Appendix A of this manuscript. The MEDLINE search strategy was adapted to enable similar searches of the other relevant electronic data bases. The reference lists of relevant systematic reviews and eligible studies were hand‐searched to identify further potentially relevant studies.

### Study Selection

The citations identified were assessed for inclusion in the review using a two‐stage process. First, two reviewers independently screened all the titles and abstracts identified by the electronic searches to identify the potentially relevant articles to be retrieved. Second, full‐text copies of these studies were obtained and assessed independently by two reviewers for inclusion using the eligibility criteria outlined in Table 1. Any disagreements were resolved through discussion at each stage, and, if necessary, in consultation with a third reviewer.

**Table 1:** Eligibility criteria

Inclusion criteria (if all of the following met)	Exclusion criteria (if any of the following met)
1. Intervention was SCS (all stimulation protocols)	1. Neurostimulation intervention other than SCS
2. Comparator was placebo stimulation	2. Comparator only included an alternative active stimulation protocol or a non‐neurostimulation control
3. Study design was an RCT	3. Design/protocol paper, methodological paper, (systematic) review, meta‐analysis, commentaries/editorial
	4. Insufficient information (e.g., study only available as a conference proceeding/abstract)

RCT, randomized controlled trial; SCS, spinal cord stimulation.

### Data Extraction

A data extraction form was designed to enable data extraction relating to study author, year of publication, country where the study was conducted, study design, population, number of participants included in the analysis, intervention including frequency of stimulation (if reported), details on placebo or sham comparator, duration of placebo or sham, patient position when programming the SCS, if an IPG programmer was available to the participants and, where applicable, consideration of carryover effects and washout periods (i.e., crossover RCTs).

Data extraction was performed by one reviewer and checked for accuracy by a second reviewer. Any disagreements were resolved through discussion, and, if necessary, in consultation with a third reviewer.

### Data Synthesis

Given the heterogeneity in patient indications and mix of parallel group and crossover RCT study designs, we did not consider it appropriate to undertake a meta‐analysis of study outcomes. Instead, a detailed narrative synthesis and structured tables were used to present the main findings from the included RCTs.

## RESULTS

### Study Selection

**Figure 1:**
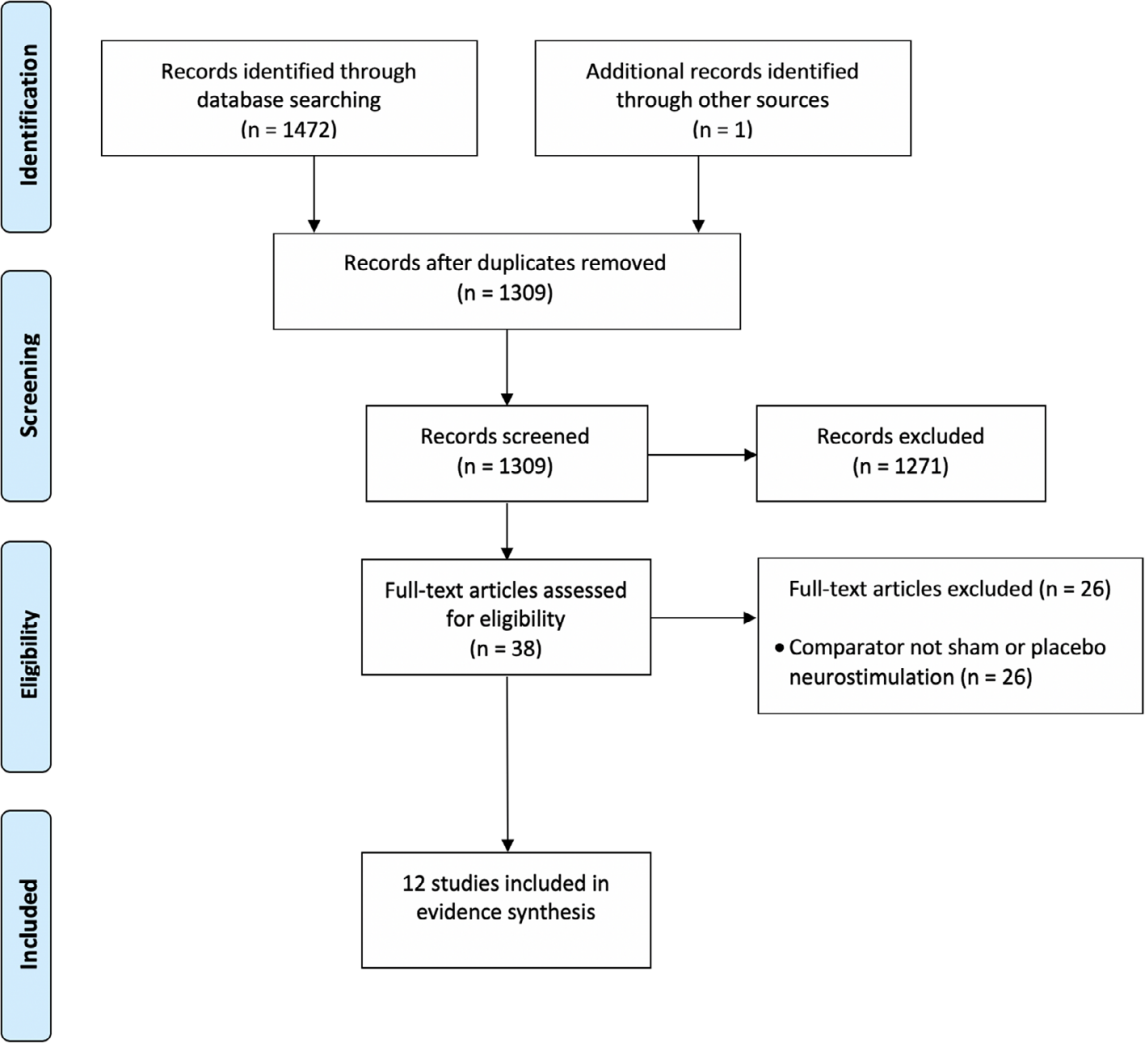
PRISMA flow chart. [Color figure can be viewed at http://wileyonlinelibrary.com]

The searches resulted in the identification of 1473 citations. After the removal of duplicate records, we identified 1309 potential citations. Following initial screening of titles and abstracts, 38 publications were considered to be potentially relevant and were retrieved to allow assessment of the full‐text publication. After review of the full‐text publications, 12 studies were included in the review 15, 16, 19, 20, 21, 22, 23, 24, 25, 26, 27, 28. Twenty‐six studies were excluded at the full‐text paper screening stage because the comparator was not a placebo or sham neurostimulation 6, 7, 8, 10, 14, 29, 30, 31, 32, 33, 34, 35, 36, 37, 38, 39, 40, 41, 42, 43, 44, 45, 46, 47, 48, 49. The PRISMA flow chart detailing the screening process for the review is shown in Figure 1.

### Characteristics of Included Studies

The characteristics of the 12 included studies are summarized in Table 2. Ten of the included studies were crossover RCTs 15, 16, 19, 20, 21, 23, 24, 25, 26, 28, while two studies were parallel RCTs, one with two arms 27 and the other with three arms 22. Eight of the studies were reported by the study authors as double blind 15, 16, 19, 21, 23, 24, 25, 28, two were single blind 22, 27, and two were unblinded RCTs 20, 26. Some studies restricted the participants to a specific condition such as FBSS 15, 16, 23, CRPS 21, or RA 20, 22, 27. Five studies included participants with a range of conditions 19, 24, 25, 26, 28.

**Table 2:** Characteristics of RCTs included

Study	Country	Study design*	Population	Number in analysis and mean age ± SD (unless otherwise stated)	Intervention	Placebo
Al‐Kaisy 15	UK	Single center double‐blind crossover	FBSS	24 (M = 16; F = 8) 47.9 years (range 33–60)	1200, 3030, and 5882 Hz	IPG turned on and discharging, but without electricity transmitted to the lead
De Ridder 19	Belgium	Single center double‐blind crossover	FBSS, FNSS, myelopathy and myelomalacia	15 (M = 4; F = 11) 54 years (range 39–68)	Burst and paraesthesia stimulation (40 or 50 Hz)	Burst stimulation was applied on the predefined electrode contacts until the patient experienced paraesthesia. Subsequently the stimulator intensity was decreased like in burst programming but continued until zero amplitude
Eddicks 20	Germany	Single center crossover	RA	12 (M = 8; F = 4) 65 ± 8 years	Paraesthesia stimulation (3 × 2 hour/day or 24 hour/day 75–85 Hz) and subthreshold (2.1–4 V)	0.1 V (thought to have no effect on the neuronal system and accordingly served as placebo)
Kriek 21	The Netherlands	Multicenter double‐blind crossover	CRPS	29 (M = 4; F = 25) 42.55 ± 12.83 years	40, 500, 1200 Hz and burst	Programming was performed with a 100 Hz stimulus to maintain an equal programming paradigm and sensation for the patient. The IPG was switched off immediately after programming and remained switched off during the two‐week test period
Lanza 22	Italy	Multicenter single‐blind three‐arm parallel group	RA	25 (M = 19; F = 6) 70.5 ± 12 years (placebo only)	1) paraesthesia stimulation and 2) subthreshold (current intensity 75–80%)	One hour of SCS every day at a current intensity of 0.05 mV
Meier 28	Denmark	Single center double‐blind crossover	CRPS and PN	14 (M = 5; F = 9) 53 years (median)	Paraesthesia stimulation	Device switched off
Perruchoud 16	Switzerland and UK	Multicenter double‐blind crossover	FBSS	33 (M = 16; F = 17) 54.2 ± 10.7 years	HF at 5 kHz	Programming occurred as for HF but the stimulator was switched off after completing programming
Schu 23	Germany	Single center double‐blind crossover	FBSS	20 (M = 7; F = 13) 58.6 ± 10.2 years	Subthreshold (500 Hz) and burst	No stimulation was programmed (device switched off)
Tjepkema‐Cloostermans 24	The Netherlands	Single center double‐blind crossover	FBSS, PN, DNP, MS, and CRPS	40 (M = 24; F = 16) 58 years (range 41–73)	Burst	Low amplitude burst (0.1 mA bursts)
Wolter 25	Germany	Single center double‐blind crossover	FBSS, CRPS, brachial plexopathy, chronic cervicobrachialgia and ulnar neuropathy	10 (M = 6; F = 4) 54 ± 6.2 years	Subthreshold	Device switched off
Youn 26	USA	Single center crossover	FBSS, RSD, migraines, and neuritis	20 (M = 4; F = 16) 52 years (range 30–80)	Paraesthesia stimulation and HF (200–1200 Hz)	Device switched off
Zipes 27	USA	Multicenter single‐blind parallel group RCT	RA	68 (M = 50; F = 18) 61 years	Paraesthesia stimulation (minimum of two hours, four times per day and as needed)	Low stimulation (above paraesthesia threshold 1 min per day)

CRPS, complex regional pain syndrome; DNP, diabetic neuropathic pain; F, female; FBSS, failed back surgery syndrome; FNSS, failed neck surgery syndrome; HF, higher frequency; IPG, implantable pulse generator; M, male; MS, multiple sclerosis; PN, peripheral neuropathy; RA, refractory angina; RCT, randomized controlled trial; RSD, reflex sympathetic dystrophy; SD, standard deviation.

* The terms single and double‐blind are presented as reported by the authors.

The type of stimulation investigated in the studies included paraesthesia inducing stimulation, subthreshold, burst, and high‐frequency SCS. Four studies included patients new to SCS (i.e., study was carried out immediately after implantation of the device) 15, 19, 22, 27. One of the studies with patients new to SCS was conducted with an external IPG system via externalized extension wires during the screening stage prior to implantation of the SCS device 19. This RCT was conducted entirely during the screening period thereby making the methodology much simpler. The remaining eight studies included patients already receiving paraesthesia inducing stimulation for at least four weeks before enrolment in the trial 16, 20, 21, 23, 24, 25, 26, 28. The phases (i.e., different settings) in the crossover RCTs ranged from two to five phases.

### Features of Placebo Comparator

The characteristics of the placebo stimulation are presented in Table 2. In one unblinded study 26 and one double‐blinded study 28, the device was simply switched off. In four studies, the device was switched off after identifying perception thresholds 25, after evoking a brief paraesthesia response during programming 23, or after completing the programming in a similar way to the intervention arm 16, 21. In one study, the amplitude was set for the sham in the same manner as for the active intervention, the IPG was on and discharging but without electricity being transmitted to the lead 15. In four studies, the device was programmed at low intensities not expected to have therapeutic effects 20, 22, 24, 27. One study named low‐amplitude burst in the publication 24, however, this was labeled as sham in the registered protocol 50. Sham was enabled in one study by first applying burst until the patient experienced paraesthesia and subsequently decreasing the stimulation amplitude to zero 19.

The types of placebo are detailed in Table 3. The duration of the placebo ranged from one week in three studies 19, 23, 25 to six months in one study 27. In one study, the device was switched off just for enough time to carry out quantitative sensory testing (QST) including a 15‐min washout period 26, while another study included a 12‐hour interval before QST assessment 28. The sham period in one study with RA patients was initially set to three months, however, after the first two patients randomized to sham stimulation were still severely symptomatic after the first month, it was considered unethical to prolong the duration of sham to more than one month 22. After one month, patients in the sham group were randomized to paraesthesia stimulation or subthreshold SCS 22.

**Table 3:** Methods of placebo

Study	Timing of study	Duration of placebo	Patient position during programming	Handheld programmer	Blinding of patients	Assessment of fidelity of blinding
Al‐Kaisy 15	Four weeks after implantation of IPG (recovery period without any active stimulation)	Three weeks (12 week crossover with four phases/different settings)	Supine	Programmer not provided to patient	Use of same programming procedure	NR
De Ridder 19	During SCS screening trial	One week (three week crossover with three phases/different settings)	Supine	Unclear	Use of same programming procedure	NR
Eddicks 20	At least three months after implantation but not >six months	Four weeks (20 week crossover with four phases/different settings)	NR	NA (patients unblinded)	NA (patients unblinded)	NA (patients unblinded)
Kriek 21	Three months after implantation	Two weeks (10‐week crossover with five phases/different settings)	Supine	Unclear	Use of same programming procedure	NR
Lanza 22	Immediately after implantation	One month	NR	Unclear	Unclear	NR
Meier 28	At least three months after implantation and an initially reported beneficial effect	12 hours (two day crossover with two phases/different settings)	NR	Unclear	SCS settings were adjusted by an assistant and were blinded to both the patient and the examiner	All but one patient were able to identify if the stimulator was ON or OFF
Perruchoud 16	Patients already treated with SCS with stable pain control	Two weeks (eight‐week crossover with two phases/different settings; before and after the first HF or sham phase there was a two‐week period with paraesthesia stimulation)	Supine	Access to programmer during washout period only. Custom‐made on/off only programmer for emergency use	Use of same programming procedure and current leak programmed during the sham periods	Fidelity of blinding confirmed
Schu 23	At least three months after implantation and patients with stable medication for at least four weeks	One week (three‐week crossover with three phases/different settings)	Sitting and supine	Programmer not provided to patient	Brief paraesthesia response during programming	NR
Tjepkema‐Cloostermans 24	At least six months after implantation	Two weeks (six‐week crossover with two phases/different settings; two‐week period with paraesthesia stimulation between the two different settings)	NR	Access to programmer during washout period only	Unclear	NR
Wolter 25	At least three months after implantation with good pain relief	One week (two‐week crossover with two phases/different settings)	Standing, sitting, and supine	Patient programmer placed in a sealed envelope available for use for unbearable pain or if patient wished to withdraw from the study	Use of same programming procedure	NR
Youn 26	Four weeks to four months after implantation	Unclear (crossover with three phases/different settings)	NR	NA (patients unblinded)	NA (patients unblinded)	NA (patients unblinded)
Zipes 27	Immediately after implantation	Six months	NR	Programmer not provided to patients randomized to placebo	Patients felt paraesthesia at a level considered insufficient to have a therapeutic effect	NR

HF, higher frequency; IPG, implantable pulse generator; NA, not applicable; NR, not reported; SCS, spinal cord stimulation.

Programming of the device was carried out in supine position in four studies 15, 16, 19, 21, sitting and supine position in one study 23, and standing, sitting, and supine positions in one study 25. Six studies did not report the patient position during programming of the device 20, 22, 24, 26, 27, 28.

In three studies, the patients were not provided with a programmer during the study period 15, 16, 23, however, in two crossover RCTs, the patients only had access to a programmer during a washout period 16, 24. It is unclear if a patient programmer was available in four of the studies 19, 21, 22, 28. In a parallel RCT, only those randomized to the intervention received a handheld programmer while those randomized to placebo (low stimulation) did not receive a programmer and therefore were not able to adjust or self‐administer SCS 27. One study mentioned that patients could switch their stimulator off in an emergency using the charging head for those with rechargeable devices and a custom‐made on/off only programmer for primary cell devices 16. For one study, the patient programmer was placed in a sealed envelope and patients were instructed to only open the envelope and use their stimulator in case of unbearable pain or if they wanted to withdraw from the study 25.

In the eight double‐blind RCTs, it is not always clear how blinding was enabled besides not providing the handheld programmer. Some studies report using the same programming procedure 15, 16, 19, 21, 25. One study stated that during programming a brief paraesthesia response was evoked in all patients in order to maintain blinding 23. In a parallel RCT, the patients in the sham arm felt paraesthesia in order to maintain blinding, but at a level considered insufficient to have a therapeutic effect 27. In a crossover RCT, to avoid unblinding patients with rechargeable devices, a current leak was programmed during the sham periods so that the recharging time and frequency were equivalent during the different crossover periods 16. Only two double‐blind crossover RCTs assessed the effectiveness of their blinding by asking participants to guess the group to which they were allocated. One study stated that all but one patient were able to identify during the study if their stimulator was turned ON or OFF, which meant that the study was actually a single‐blind RCT 28. The other study observed proportions of patients guessing correctly that can be expected from chance with 45% guessing correctly at visit 3 and 55% at visit 5 16.

Four of the crossover RCTs did not consider a washout period between the different stimulation phases 15, 19, 23, 25. In the studies that included a washout period, the period consisted of 15‐min 26, 12‐hour 28, two‐day 21, or a two‐week washout period with their own paraesthesia stimulation 16, 24. One study included one‐week wash‐in period 20.

## DISCUSSION

The recent development of paraesthesia free SCS approaches has resulted in a growing number of RCTs evaluating SCS compared to a placebo control. In the 12 RCTs identified in this systematic review, the placebo varied from simply switching off the SCS device to more complex approaches such as intermittent switch on of low current stimulation or programming a current leak during the placebo periods so that the recharging time and frequency were equivalent during the different crossover periods. The nature of the placebo may affect the validity and replicability of RCT findings.

The reporting of the methods to enable placebo is highly variable and some authors omitted key information to interpret validity such as whether patients were provided with a handheld programmer for the duration of the study or not 23, 26. Similarly, studies failed to report the position of the patient when programming a device for a subperception threshold comparator and the subsequent sham arm. The position of the patient at this point is important for the threshold establishment in subthreshold stimulation because thresholds are about 25% higher in upright than supine positions, and thus postural changes can lead to exceeding perceptual threshold 51. Additionally, in patients where no threshold is detected, a predefined strategy is needed for dealing with that eventuality.

It is possible that initial stimulation may produce a prolonged effect and that the presence or duration of a carryover effect of SCS has not been fully established. A period effect may also be observed where the first modality produces a higher magnitude of effect regardless of its nature.

We believe this article to be the first systematic review of placebo control methods in RCTs of SCS. The review process, including study identification, selection, and data extraction, was carried out in line with PRISMA 18 and CRD guidance 17. However, we did not assess the quality/risk of bias of the included studies. The aim of this review was to describe the different methods used to enable a placebo comparator arm in RCTs of SCS and not to report on the findings of the included RCTs or the validity of the findings.

Authors of future SCS placebo‐controlled studies should consider a number of specific aspects of the design and reporting on their trial (Table 4). For studies using non‐rechargeable devices, the following needs to be reported: programming parameters for the active and the sham arm, how the patient handheld programmer was managed and if a handheld programmer was provided to the patients, how was blinding ensured. Studies that utilize subthreshold programming as a comparator need to specify the position(s) in which the threshold was measured in and whether a feedback loop/position adjustment was utilized to modulate current intensity. The duration of daily use and frequency of programmer interactions should also be reported. Trials that seek to compare subthreshold stimulation from different manufacturers with a placebo comparator arm should consider the feasibility of blinding, as the research team and patient may be aware of logos associated with the different manufacturers as well as access to manufacturer website information. For rechargeable devices, the use of placebos is further compounded by a number of factors, including the need for the patients in both arms to experience a similar recharging burden. Accordingly, the frequency and duration of recharging should be reported. This is important in both crossover and parallel design studies. A current leak therefore needs to be programmed into the IPG of the placebo group of a similar magnitude to the current flow in the active arm, or the recharger needs to be modified. Perruchoud et al 16 and Al‐Kaisy et al 15 reported a current leak from the IPG equivalent to the calculated current consumption in the subthreshold groups based on current setting and fixed values for pulse width and frequency. The same is not possible where pulse width and intensity values are varied between groups such as in Schu et al. 23. The management of the patient handheld programmer needs to be specified and if withheld, researchers need to state what provision was made for subjects to switch off their SCS in an emergency. Finally, the management of the patient recharger needs to be specified, particularly where the recharger contains a feedback screen that allows the subject to assess IPG charge.

**Table 4:** SCS placebo checklist—Items to include when reporting trials of SCS including a placebo arm

Item	Recommendation
Programming and management when the study includes patients with non‐rechargeable devices	Report programming parameters for the active and the sham arm
	Describe how the patient handheld programmer was managed
	State how blinding was ensured if the patient handheld programmer was provided to the patients
	For studies that utilize a subthreshold programming as a comparator:
	Identify the position that the threshold was measured in
	State if a feedback loop/position adjustment was utilized to vary current with position
	Report the duration of daily use and frequency of programmer interactions
Programming and management when the study includes patients with rechargeable devices	Describe how a similar recharging burden was ensured in the different arms (i.e., report the frequency and duration of recharging)
	Report how the patient handheld programmer was managed (particularly if it contains a feedback screen that allows the subject to assess IPG charge)
	Describe what provision was made for subjects to switch off their SCS in an emergency if patient handheld programmer was withheld
Research team	State if the team was split into blinded and unblinded side with no intermixing
	Report if there was one unblinded programmer member of the team
	Clearly state which members of the research team were blinded
Effectiveness of blinding	Describe how effectiveness of blinding of patients and members of the research team was assessed
Sham sensations	Describe how sham sensations were managed

IPG, implantable pulse generator; SCS, spinal cord stimulation.

There are several possibilities to manage sensations related to placebo responses depending on the nature of the active comparator. These include:

1. Devices that cyclically switch on to deliver a short burst of suprathreshold stimulation. However, even this minimal “dose” might be therapeutic.
2. Devices that deliver subthreshold current of very low intensity continuously or intermittently. This too might be therapeutic.
3. Devices that are fully switched off. Only this strategy avoids the risk that stimulation might be therapeutic, even when the dose is minimal. Use of a full switch off strategy against a paraesthesia stimulation comparator risks unblinding participants.

Other issues related to study design of placebo‐controlled SCS trials are common to RCTs in other areas. If the RCT is double‐blinded the investigating team should be clearly split into blinded and unblinded sides with no crossover. The members of the investigating team who are blinded should be clearly stated, including outcome assessors. A single unblinded member of the team should perform device programming where possible to ensure consistency. Ideally the unblinded programmer should not have conflicts of interest and follow a similar “script” in both arms. Consistent training in programming or standard programming sequences should be made available in multicenter studies to ensure consistency in programming across study sites, particularly of the sham arm where programming duration may be significantly shorter than other modalities. Researchers of sham‐controlled studies are urged to assess the effectiveness of their blinding by asking participants to guess the group to which they were allocated. Researchers of sham‐controlled studies should also assess patients' expectation of benefit before starting the trial and perception of effectiveness at the end of the trial 52.

Despite not being particular to SCS placebo‐controlled studies, it is an ethical requirement to include a pain management plan to manage study participants' pain. Participants are informed that they have the right to exit any study at any time. It is important that subject information be managed, and participant interaction during parallel design studies should be minimized.

In conclusion, with the development of new stimulation protocols there has been an increase in the number of placebo‐controlled RCTs of SCS. The methods to achieve sham and blinding of patients are not always clearly described which may lead to concerns about the validity and replicability of the findings. We provide recommendations on the design and reporting of future placebo‐controlled RCTs in the field of SCS.

## Authorship Statements

Sam Eldabe conceptualized the study. Ewan McNicol conducted the searches. Rui Duarte, Ewan McNicol, and Sam Eldabe screened the search results for eligibility. Rui Duarte and Sam Eldabe extracted the data. All authors contributed to drafts of the manuscript and approved the final version of the manuscript.

## APPENDIX A: MEDLINE SEARCH STRATEGY 1

1. spinal cord stimulat$.ti,ab,kw.
2. dorsal column stimulat$.ti,ab,kw.
3. epidural stimulat$.ti,ab,kw.
4. or/1–3
5. exp PAIN/
6. pain*.mp.
7. (neuralgi* or myalgi* or neuropath* or arthriti* or osteoarthri* or arthralgi* or sciatica or headache* or migrain*).mp.
8. exp ANALGESIA/
9. analgesi*.mp.
10. exp Tibial Neuropathy/ or exp Femoral Neuropathy/ or exp Radial Neuropathy/ or exp Alcoholic Neuropathy/ or exp Optic Neuropathy, Ischemic/ or exp Median Neuropathy/ or exp Sciatic Neuropathy/
11. Critical limb ischemia.kw.
12. lower limb ischemia.kw.
13. leg ischemia.kw.
14. exp ATHEROSCLEROSIS/
15. exp Vascular Diseases/ or exp Peripheral Vascular Diseases/ or exp Peripheral Arterial Disease/ or exp Arteriosclerosis/ or exp Ischemia/ or exp Arterial Occlusive Diseases/
16. or/5–15
17. randomized controlled trial.pt.
18. controlled clinical trial.pt.
19. randomized.ab.
20. placebo.ab.
21. drug therapy.fs.
22. randomly.ab.
23. trial.ab.
24. groups.ab.
25. or/17–24
26. (animals not (humans and animals)).sh.
27. 25 not 26
28. 4 and 16 and 27
